# The disengagement of visual attention in Alzheimer's disease: a longitudinal eye-tracking study

**DOI:** 10.3389/fnagi.2015.00118

**Published:** 2015-06-23

**Authors:** Trevor J. Crawford, Alex Devereaux, Steve Higham, Claire Kelly

**Affiliations:** Dementia Research and Eye Tracking Lab, Department of Psychology, Centre of Aging Research, Centre for Learning and Human Development, Lancaster UniversityLancaster, UK

**Keywords:** dementia, eye-tracking, Alzheimer's disease, attention, cognition

## Abstract

**Introduction:** Eye tracking provides a convenient and promising biological marker of cognitive impairment in patients with neurodegenerative disease. Here we report a longitudinal study of saccadic eye movements in a sample of patients with Alzheimer's disease and elderly control participants who were assessed at the start of the study and followed up 12-months later.

**Methods:** Eye movements were measured in the standard gap and overlap paradigms, to examine the longitudinal trends in the ability to disengage attention from a visual target.

**Results:** Overall patients with Alzheimer's disease had slower reaction times than the control group. However, after 12-months, both groups showed faster and comparable reductions in reaction times to the gap, compared to the overlap stimulus. Interestingly, there was a general improvement for both groups with more accurately directed saccades and speeding of reaction times after 12-months.

**Conclusions:** These findings point to the value of longer-term studies and follow-up assessment to ascertain the effects of dementia on oculomotor control.

## Introduction

Alzheimer's Disease (AD) is a major cognitive disorder of older adults that blights the lives of millions of people and their families across the globe (Stokes, 2013). Many sufferers are undiagnosed due to the lengthy clinical and psychometric procedures that are often used by local and national health services. Eye tracking provides a convenient and promising biological marker of cognitive impairment in patients with neurodegenerative disease (Crawford et al., 2013), and is likely to enhance the current procedures for early diagnosis and long-term monitoring of disease progression. Comprehensive studies on the profile of eye movement control in dementia are essential in order to fully exploit its full potential.

Clinicians and researchers have tended to focus on the problems in memory retrieval, which may occur relatively late in the evolution of AD. However, there is increasing evidence that there are subtle impairments in visual attention and other cognitive domains that occur early in the course of the disease. Several studies have reported a dysfunction in the disengagement of attention in AD (Della Sala et al., 1992; Parasuraman et al., 1992; Parasuraman and Haxby, 1993; Scinto et al., 1994; Perry and Hodges, 1999; Baddeley et al., 2001; Solfrizzi et al., 2002; Tales et al., 2002) which appears to coincide with the progressive decline in working memory and executive function (Awh and Jonides, 1998; Parasuraman and Greenwood, 1998). Parasuraman et al. (1992) reported that AD patients, of mild severity, displayed an attention-shifting or disengagement deficit in a letter-discrimination task, in a similar way to patients with hemi-neglect that was caused by a lesion in the parietal lobe (Posner et al., 1984). In AD, the speeding-up of response times to a “valid” cue (i.e., a cue that signaled the correct location of the target), did not differ from healthy controls. In contrast, the slowing-down of response times following an “invalid” cue (i.e., a cue that signaled an incorrect location of the target), was abnormally high, compared to healthy controls. This implied that attention to spatial locations was preserved in early AD, whereas the ability to disengage (or “unplug”) attention was impaired. Using PET Parasuraman et al. (1992) also reported that the degrees of disengagement deficit was correlated with the level of hypo-metabolism in the superior parietal lobe.

**Figure 1:**
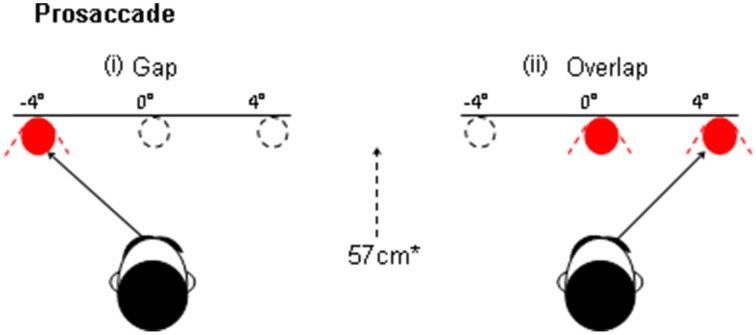
**An illustration of the “gap” and overlap paradigms**. In the gap task the fixation point is withdrawn prior to the presentation of the peripheral target. In the Overlap paradigm the fixation remains on at the onset of the target.

**Figure 2:**
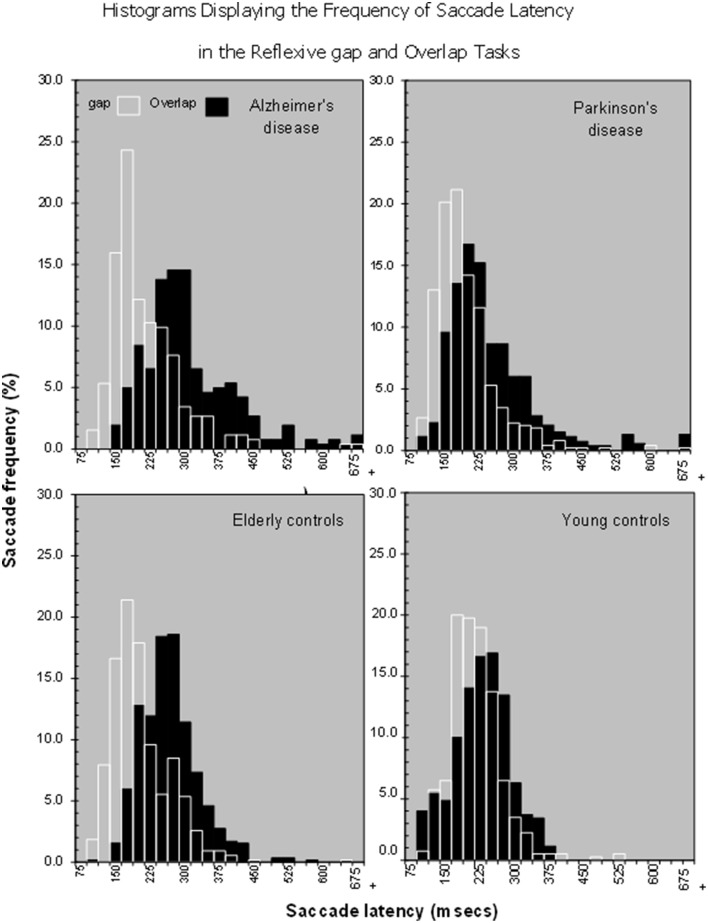
**Histograms showing that there is a shift in the distribution of saccade latencies in the overlap stimuli in comparison to the gap stimuli in various patient groups, Alzheimer's Disease, Parkinson's disease, Older and young controls**.

The process of disengagement has also been widely investigated using the gap and overlap saccadic paradigms (Saslow, 1967; Fischer and Boch, 1983; Kalesnykas and Hallett, 1987; Braun and Breitmeyer, 1988; Fischer and Weber, 1993). These paradigms have also been explored in newborns (Farroni et al., 1999) and non-human species (Kano et al., 2011). In a traditional “Gap” paradigm (Figure 1) the onset of the peripheral target is preceded by a short period (usually 200 ms), when the current fixation point is switched-off, leaving a brief “gap” between the offset of the fixation point and the onset of the target for the saccade. In the “overlap” paradigm, the fixation point remains on for a period of time, when the peripheral target is presented. The gap paradigm facilitates the disengagement of attention from the fixation point and therefore yields faster saccadic response times, since there is no other visual stimulus to compete with the target. The overlap slows the disengagement of attention since the persistence of the fixation point continues to capture attention whilst the target is presented (see Figure 2, based on Crawford et al., 2013). The ability to “unplug” visual attention can be readily estimated by measuring the time it takes for the eye to begin a saccadic eye movement towards the gap stimulus, in relation to the overlap paradigm. According to one widely supported scheme (Findlay and Walker, 1999), the gap manipulation helps to resolve the competition that occurs between two mutually exclusive activities: the eye fixation and saccade initiation systems. One important physiological arena for this competition is the superior colliculus (Munoz and Wurtz, 1993a,b; Dorris and Munoz, 1995; Dorris et al., 1997). Top-down saccadic eye movement signals are also controlled by various cortical areas including the parietal and frontal cortex (Dias and Bruce, 1994; Hanes and Schall, 1996; Müri et al., 1998; Munoz and Everling, 2004).

Yang et al. (2013) found that the “gap” effect was increased in patients with mild cognitive impairment and mild to moderate Alzheimer's disease, suggesting a potential biological marker for AD. This implies that they have difficulty in unplugging, or transferring attention away from events. However, no study has yet examined the longitudinal effects of the disease on eye movements in these paradigms. Therefore, we examined the hypothesis that the magnitude of the “gap” effect will increase over time, due to a deterioration in the ability to disengage attention. An alternative outcome is that the magnitude of the “gap” effect will not increase, but that there will be a proportional change in saccade latency for both the gap and overlap paradigms. This would result in a “gap” effect that remains relatively stable across the disease, showing that this effect changes with age, rather than the disease (Crawford et al., 2013).

## Materials and methods

### Participants

Elderly control participants were volunteers from the local community (*N* = 25; age range = 62–80 years; mean = 70.6; *SD* = 4.9; male *n* = 8; female *n* = 17; see Table 1). The AD group comprised mild-to-moderate patients with Alzheimer's disease recruited from the Dementia Research Project at Lytham Hospital Memory Clinic, United Kingdom. All patients fulfilled the criteria for probable Alzheimer's disease according to the American Psychiatric Association's DSM IV (American Psychiatric Association, 2000) and the National Institute of Neurological and Communicative Disorders and Stroke (NINCDS) (*N* = 11; age range = 71–88; mean = 78; *SD* = 4.94; male *n* = 6; female *n* = 5, see Table 2). Three patients from the original sample with a diagnosis of vascular or mixed dementia were not included in these analyses. All patients underwent a detailed clinical history, physical/neurological examination and routine investigations: hemoglobin, full blood count, erythrocyte sedimentation rate, urea and electrolytes, liver function tests, blood glucose, thyroid function tests, serum vitamin B12, and folate, serology for syphilis and urinalysis. Cognitive impairment was assessed with the Standardized Mini Mental State Examination (SMMSE) (Folstein et al., 1975; Molloy et al., 1991) and the cognitive sub-scale of the Alzheimer's Disease Assessment Scale (European version; EADAS-cog); (Rosen et al., 1984; Dahalke et al., 1992). Dementia severity was conducted at baseline using the Clinical Dementia Rating Scale (CDR) (Hughes et al., 1982). All participants underwent a detailed neuropsychological assessment (see Crawford et al., 2013), [National Adult Reading Test (NART) (Nelson, 1982), Verbal Fluency (Storandt et al., 1984), Trail Making Form A and B (Reitan, 1958), Digit Span from Wechsler Adult Intelligence Scale III (Wechsler, 1997a), Spatial Span from Wechsler Memory Scale III (Wechsler, 1997b) and the Gibson Spiral Maze (Pattie and Gilleard, 1987)]. All participants were right-handed, with normal or corrected Snellen chart visual acuity. No participant demonstrated visual neglect on the line bisection task (Schenkenberg et al., 1980). Written informed consent was obtained from all participants after a detailed description of the study, which was approved by the Blackpool, Wyre, and Fylde Local Research Ethics Committee.

**Table 1:** **Demographics and cognitive assessment at baseline and at the 12-month (12) follow-up in the control group**.

**Controls**	**Age**	**MMSE (12)**	**EADAS (12)**	**Years ED**
1	65	30(29)	5(4)	12
2	67	29(28)	11(5)	14
3	65	30(29)	4(6)	10
4	65	27(29)	9(6)	10
5	62	29(30)	5(2)	13
6	78	30(29)	11(8)	15
7	71	30(29)	5(6)	12
8	65	29(29)	8(2)	13
9	68	30(30)	9(5)	20
10	76	28(30)	7(8)	12
11	63	30(30)	3(4)	12
12	73	30(30)	9(5)	12
13	69	28(29)	11(5)	17
14	68	30(30)	7(4)	12
15	76	30(30)	9(6)	12
16	71	29(30)	8(0)	12
17	71	30(27)	10(9)	9
18	74	29(29)	7(5)	14
19	76	30(28)	8(11)	11
20	77	27(28)	8(7)	9
21	68	30(30)	6(4)	17
22	71	28(30)	12(8)	10
23	80	30(27)	10(7)	10
24	73	30(30)	4(6)	12
25	73	30(30)	7(4)	9
Mean	70.60	29.3(29.4)	7.7((5)	12.4
*SD*	4.97	0.99(0.6)	2.5(1.8)	2.7

**Table 2:** **Demographics and cognitive assessment at baseline and at the 12-month (12) follow-up in the Alzheimer group**.

**Dementia**	**Age**	**MMSE(12)**	**EADAS(12)**	**Years ED**
1	71	27(26)	11(10)	12
2	88	23(22)	22(23)	9
3	77	27(27)	17(21)	11
4	76	21(20)	21(25)	12
5	80	24(25)	23(19)	9
6	78	20(27)	16(19)	9
7	75	23(13)	26(28)	10
8	84	16(9)	39(44)	14
9	80	27(30)	12(12)	10
10	72	23(21)	22(18)	14
11	77	29(25)	10(11)	12
Mean	78.0	23.64(22.27)	19.91(20.09)	11.09
*SD*	4.94	3.78(6.34)	8.28(10.12)	1.87

#### Assessment of saccadic eye movements

Saccadic eye movements were recorded using an infra-red scleral reflection eye-tracker “ExpressEye” (Optom, Freiburg, Germany). The eyes were recorded monocularly with a temporal resolution of 1 ms and spatial resolution of 0.1°. The system is linear over a 15° visual field. The stimulus display presented a central fixation point within an unfilled 0.75 × 0.75° empty square marker; the target was a red 0.4° spot, which was projected left and right horizontally. The device projected these stimuli from a head-mounted laser onto a white tangential screen at 57 cm. The laser output was 0.2 mW, with a wavelength of 635 nm with a luminance of 66.37 cd/m^2^. The three head-mounted lasers provided a simple way to compensate for lateral head motion.

##### Gap prosaccade paradigm

Each trial commenced with the central fixation point (see Figure 1) that was presented within a central square marker for 1000 ms. The central square remained visible throughout all of the trials and provided a useful reference point for the stabilization of the head. The fixation point was then removed for a period of 200 ms (i.e., “gap”) before the saccade target was presented at ±4° (randomized). The target was switched off for an interval of 1200 ms when only the central square was visible. The central fixation point was then presented at the start of the next trial.

##### Overlap prosaccade paradigm

An identical stimulus was used in the overlap display, where the procedure was similar to the gap display apart from the timing of the removal of the central fixation point. Here, the fixation point “overlapped” for 200 ms with the presentation of the pro-saccade target, whereas the fixation point was removed before the target was presented in the gap task described previously.

##### GO/NO–GO paradigm

At the start of each trial a central fixation light was illuminated for 1000 ms. This central light was then switched off, followed by a 200 ms “GAP.” At the termination of the “GAP” period a target was presented at ±4° for 700 ms, while the central fixation point remained off. The next trial commenced after an interval of 1200 ms during which only the central square was presented. Three versions of this paradigm examined the ability to maintain central attention and to ignore a target that was presented in the left, right or both visual fields. (A) NO-GO: Participants were instructed to ignore the target light and to maintain fixation at the center of the screen for the duration of the trial. (B) GO-RIGHT/NO-GO-LEFT: Participants were instructed to “look” at the targets that were presented in the right visual field, but to suppress eye movements to all targets in the left field. (C) GO-LEFT/NO-GO-RIGHT: Participants were required to “look” at the targets that were presented in the left field but to suppress eye movements to all targets in the right field. Inhibitory control was assessed at the baseline assessment in a subset of patients and controls.

#### Measurement of saccadic parameters

The start and end of a saccade was initially detected at the point at which the eye velocity crossed 30°/s threshold. These measurements include: the amplitudes and reaction times of the primary saccade that was generated toward or away from the target, and proportion of correctly directed saccades toward the target. Tests for homogeneity of variance were conducted using IBM SPSS Statistics 21.

## Results

### Saccade reaction times

**Figure 3:**
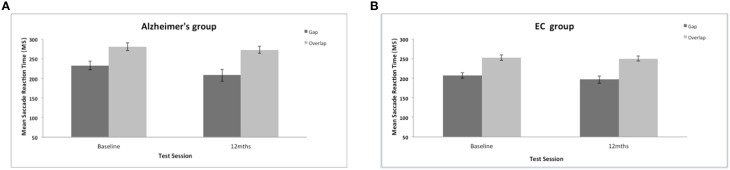
**(A,B)** Figure shows the mean saccadic reaction times (in ms, with standard error bars) to the gap and overlap stimuli at baseline and 12-months for AD patients **(A)** and elderly controls **(B)**.

The present work investigated whether or not the magnitude of the reflexive “gap” effect changes longitudinally in patients with AD. AD were slower to generate a saccadic eye movement towards the target in comparison to the control group (mean = 226 ms, *SE* = 4.97); AD group {mean = 249 ms, *SE* = 7.4; [*F*_(1, 35)_ = 6.43, *p* = 0.016]}. There was also a significant effect of test session [*F*_(1, 35)_ = 4.77, *p* = 0.036] with a general reduction of mean saccadic reaction times (RTs) at the 12-month session (see Figure 3), but with no interaction with group [*F*_(1, 38)_ = 0.739, ns]. The size of the “gap” effect was measured as the difference between the overlap and gap RTs. The “gap” effect was highly significant [*F*_(1, 35)_ = 86.45, *p* = 0.001]; overall both groups revealed slower reaction times in the overlap, compared to the gap task. Both groups benefited from the gap effect: At the baseline assessment the control group “gap” effect = 46 ms; whilst the “gap” effect for the AD patients = 48 ms; At 12-months the control group revealed a “gap” effect = 54 ms and the dementia patients showed a “gap” effect of 65 ms. There was no overall group difference in the magnitude of the “gap” effect [*F*_(1, 35)_ = 0.37, ns] and no change in the effect at 12-months [*F*_(1, 35)_ = 2.7, ns]. Clearly, the “gap” effect is relatively well preserved in patients with AD. Indeed, Yang et al. (2013) reported a large “gap” effect for AD patients (115 ms) and for the control group (88 ms). However, it is not clear whether the “gap” effect increases with age, disease progression or both (see Crawford et al., 2013).

### Saccadic amplitudes (degrees)

**Figure 4:**
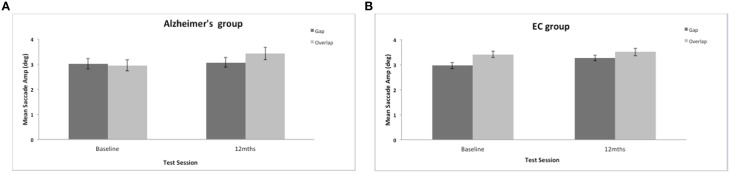
**(A,B)** Figure shows the mean saccadic amplitudes (in degrees, with standard error bars) to the gap and overlap stimuli at baseline and 12-months for AD patients **(A)** and elderly controls **(B)**.

There was a significant effect of the stimulus gap on saccadic amplitudes for both groups [*F*_(1, 34)_ = 8.3, *p* = 0.007]. Saccades were generally of larger amplitude and more accurate to the overlap stimulus (see Figure 4). There was no significant effect of group on the mean amplitude of the prosaccades [*F*_(1, 37)_ = 1.008, ns]; no effect of test session [*F*_(1, 37)_ = 2.48, ns], and no interaction between session and group status [*F*_(1, 34)_ = 0.049, ns]. In comparison to other eye movement features (see Crawford et al., 2005, 2013) the ability to modulate the amplitude of a saccade is clearly preserved well into the course of the disease.

### Saccadic direction (%correct)

**Figure 5:**
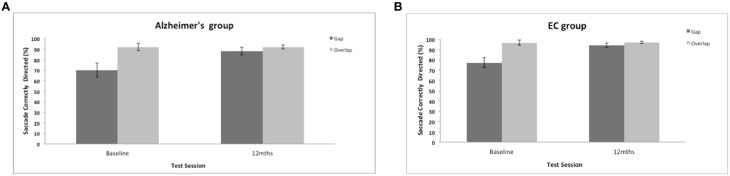
**(A,B)** Figure shows the mean frequency of the correctly directed saccades (with standard error bars) to the gap and overlap stimuli at baseline and 12-months for AD patients **(A)** and elderly controls **(B)**.

Saccadic direction to the gap and overlap targets revealed a marginal trend of a group effect [*F*_(1, 35)_ = 3.7, *p* = 0.062]. Figure 5 shows a high proportion of correctly directed saccades for the AD and control groups (overall mean = 86%, *SE* = 2.1; control mean = 91%, *SE* = 1.44). Patients did not have great difficulty aiming their eyes in the correct direction and were correct on most trials. There was a strong effect of test session [*F*_(1, 35)_ = 18.5, *p* = 0.001] with a general improvement in accuracy in both groups after 12-months. There was a strong effect of the stimulus gap [*F*_(1, 35)_ = 20.72, *p* = 0.001] for both patients and controls revealing more correctly directed eye movements towards the overlap target (Figure 5), but with no interaction with participant group or test session.

### Z-score plots

**Figure 6:**
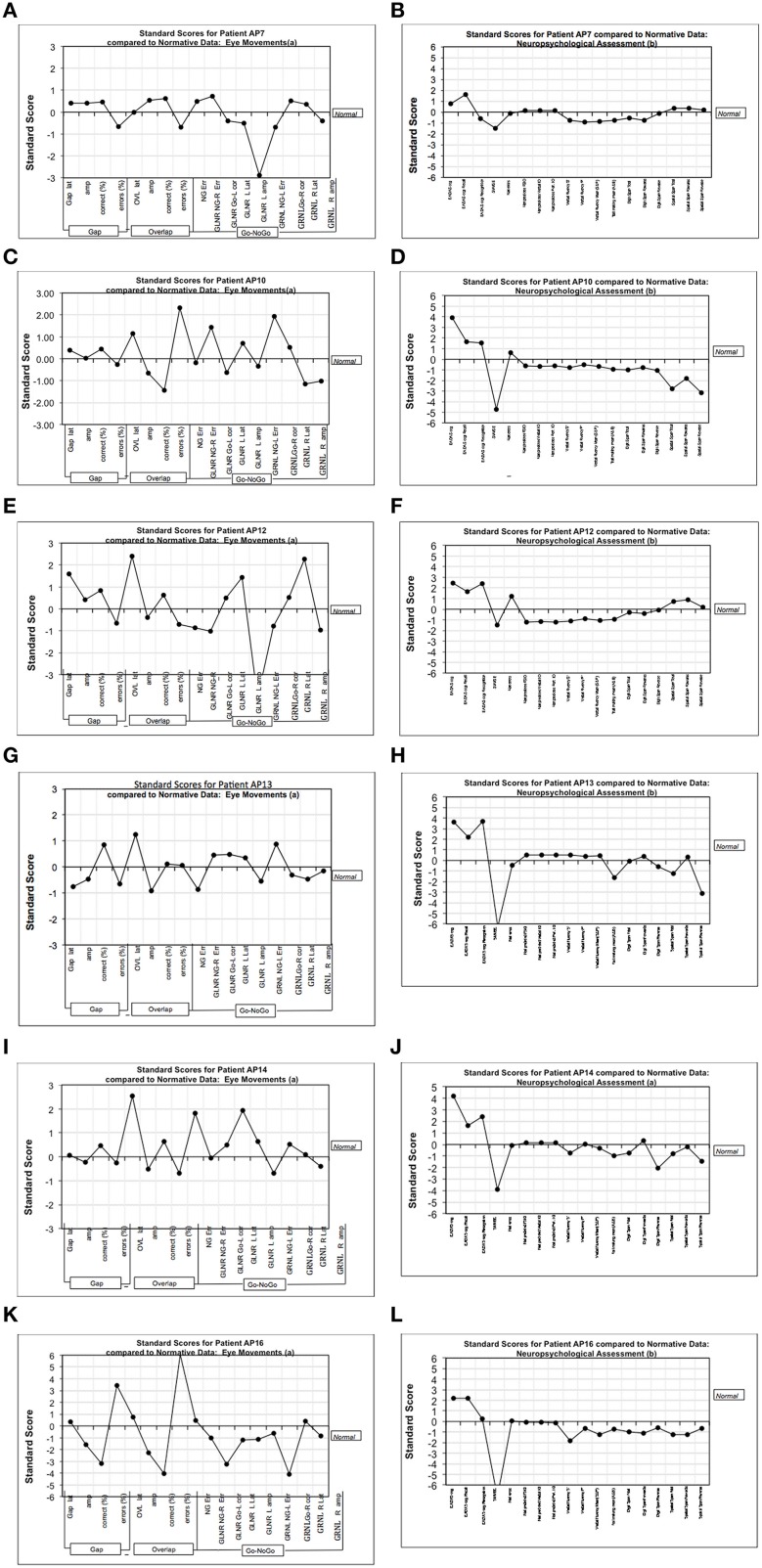
**Figure shows the chart of z-scores across cognitive assessments, together with eye-tracking z-scores**. The charts represent standardized (standard deviation) scores with reference mean of the control group (0-line). **(A,C,E,G,I,K)** Show the z-scores (with reference to the control group) from the various measures in the eye-tracking tests for six mild-moderate AD patients. **(B,D,F,H,J,L)** Show the equivalent z-scores for these AD patients using our test battery of traditional cognitive assessments. The traditional cognitive tests are generally close to the 0-line, and are relatively flat in comparison to the more sensitive measures of eye-tracking. VF “F,” Verbal fluency–letter F; VF “P,” Verbal fluency–letter P; EADAS Rec, Recall subtest; EADAS Recog, Recognition subtest; Nart predicted FSIQ, NART full scale IQ; Trails A and B, Trail making mean (A and B); DS, Digit Span; SS, Spatial span; NG, No-Go task; GLNR, Go Left, No Go Right in the Go-No-Go task; GRNL, Go Right, No Go Left in the Go-No-Go task; R, right; L, Left; NG-Err, frequency of direction errors in direction of the target; Go-L corr, frequency of correct saccades toward the target in Go-No task; Amp, saccade amplitude; Lat, saccade latency; Err, saccade direction errors.

From the perspective of clinical diagnosis it is important to supplement eye-tracking analyses that are based on group data with evidence from individual cases. Therefore, we examined the data for AD patients who were tested across the full range of oculomotor paradigms. Each saccadic parameter is expressed as a z-score ($z=\frac{\left(\bar{x}-x\right)}{SD}$) with reference to the equivalent mean score for the control group. Figure 6 shows the chart of z-scores across the cognitive assessments, together with the eye-tracking z-scores. Unsurprisingly, AD patients revealed high z-scores for the EADAS Cog and MMSE cognitive assessments, which helped to inform the clinical diagnosis. Z-scores across the battery of neuropsychological measures were typically within ±1 standard deviation of the control group (0-score reference line). For the eye-tracking assessments the patients varied in their profile of z-scores across the saccadic features. ADs differed from the controls by 2 standard deviations or more for at least one of the oculomotor parameters. This implies that oculomotor assessment in AD will benefit from a comprehensive range of oculomotor tests with reference to a standardized z-score profile, as an alternative to the more common approach that employs limited measures from a specific oculomotor paradigm.

### Cognitive assessment (MMSE and EADAS); years of education and age

A substantive level of cognitive impairment was revealed at the baseline assessment by the lower scores on the MMSE [*F*_(1, 35)_ = 50.54, *p* < 0.01] and the higher EADAS scores [*F*_(1, 35)_ = 46.94, *p* < 0.01] in the AD group. This cognitive impairment was also evident at the 12-month follow-up assessment [MMSE *F*_(1,35)_ = 29.38, *p* < 0.01; EADAS *F*_(1, 35)_ = 47.83, *p* < 0.01]. A strong correlation was revealed between the cognitive scores at baseline and at follow-up for the AD group (MMSE, *r* = 0.698, *p* = 0.017; EADAS Cog, *r* = 0.942, *p* = 0.001). At the 12-month follow-up the AD group revealed a decline in spatial memory [*t*_(10)_ = 2.472, *p* = 0.03] from the baseline assessment. There was no reliable change in the AD scores for MMSE [*t*_(10)_ = 0.986, ns], EADAS [*t*_(10)_ = −0.666, ns]. Similarly, NART errors, digit span, Trails A and B, verbal fluency, Gibson maze–all ns) all showed no change at the 12-month follow-up. Importantly, the groups were matched on years of education [*F*_(1, 35)_ = 2.005, ns]. The mean age of the control group (70.6 years), was lower than the AD group (78 years). However, there was no correlation of saccadic reaction times with age at baseline (*r* = 0.119, *p* = 0.55 ns) nor at the 12-month follow-up (*r* = 0.134, *p* = 0.397 ns). Thus, age did not predict saccadic reaction times within this cohort. To further confirm that neither age nor years of education were a predictor of performance, a further analysis of variance was conducted on a subgroup of AD patients and controls matched on mean age and years of education. The results confirmed clear effects of test session and saccade task (faster reaction times for the gap task for both groups).

## Discussion

There have been relatively few longitudinal investigations of eye movements in dementia. To our knowledge there have been only two such studies (Hutton et al., 1984; Bylsma et al., 1995). Here we report a longitudinal study with assessments at baseline and a 12-month follow-up. Yang et al. (2013) reported a larger “gap” effect in patients with AD in comparison to the controls. These longitudinal data revealed that this robust “gap” effect in AD, was maintained at a 12-month follow-up. Crawford et al. (2013) reported that the “gap” effect was larger in older groups compared to younger participants, which suggests that the “gap” effect may provide a marker of aging, rather than neurodegenerative disease. Surprisingly, the AD reaction times were selectively decreased in the gap task over the course of the assessments. It is remarkable that both groups revealed a similar change in reaction times and saccade direction at the 12-month follow-up session. Why could this be? A number of non-specific factors are likely to have contributed to the overall improvement. Saccadic eye movements toward a target (i.e., prosaccades) are a low, level visuomotor behavior that we produce hundreds of times every day. It should not be surprising that this everyday activity can improve in a laboratory setting. Initial test anxiety, is likely to be reduced with increased task familiarity and less distractibility in the research environment. What is intriguing is that these factors may also be relevant to people with mild dementia. Although speculative, it may be possible that eye-tracking paradigms can tap into a reserved capacity to respond to implicit cues that has been previously reported in patients with dementia (Gabrieli et al., 1993; Verfaellie et al., 2000; Ballesteros and Reales, 2004; Debra and Fleischman, 2007). Future work will explore this further to determine whether implicit learning is corroborated with converging evidence from additional sources.

These findings revealed that the prosaccade tasks were able to discriminate AD patients from healthy controls at baseline assessment. Z-score eye-tracking charts revealed large standardized deviations at the level of individuals with reference to the data from the control group. However, the ability to detect AD is distinct from the challenge of monitoring the progressive changes in the disease. The current evidence suggests that prosaccades, as a tool to examine attentional disengagement, may help to meet the former, but not the latter challenge. To fully explore eye movements as a tool for monitoring the progressive brain changes in AD, will require additional paradigms, that can incorporate cognitive operations, such as spatial memory (see Crawford et al., 2013). Given that spatial memory was the principal measure of decline at follow-up, it is not surprising that there was little decline in prosaccades. In contrast to other saccade tasks (e.g., antisaccades) prosaccades do not place a high cognitive load on spatial memory.

### The “gap” effect and the brain

Saccadic eye movements are generated by the reciprocal activation of saccade-related neurons and the inhibition of fixation neurons in the superior colliculus (Munoz and Wurtz, 1993a,b; Dorris and Munoz, 1995; Dorris et al., 1997). According to the Findlay and Walker (1999) model, this process is enhanced in the “gap” task. Switching off the fixation point reduces the activation of the fixation cells (i.e., the equivalent of releasing the brakes in a car), and releases the movement cells from inhibition. By reducing the activity of the fixation cells, the offset of the fixation point, therefore shortens the reaction time of the saccadic eye movement (Dorris and Munoz, 1995). Conversely, when the fixation point remains on (as in the overlap condition), disengagement will be delayed, due to the activation of the fixation cells and the inhibition of the movement cells.

Event related potential (ERP) studies have revealed that a number of neural correlates contribute to the gap effect, including preparatory neural activity in prefrontal cortex, increased visual cortical activity on gap trials and increased parietal activity in the overlap condition (Csibra et al., 1997). Kawakubu et al. (2002) discovered that target-locked ERPs in the gap task facilitated attentional disengagement, some 60 ms prior to the onset of the target stimulus. Saccade-locked ERPs revealed that pre-saccadic activity was greater in the overlap condition, compared to the gap condition. FEF appears to be critically involved in the generation of volitional saccades and in the disengagement of fixation (Rivaud et al., 1994; Pierrot-Deseilligny et al., 2004). In the saccade overlap task, saccadic latencies were increased in patients with a lesion that incorporated FEF (Rivaud et al., 1994). However, in the gap paradigm saccadic latency was unaffected in these patients (Pierrot-Deseilligny, 1991; Rivaud et al., 1994). Patients with lesions of the PPC showed a bilateral increase of the latencies of saccades in the gap task (Pierrot-Deseilligny et al., 1987, 1991), while latency is even more prolonged in the overlap task (Walker and Findlay, 1996). The selective effects in the overlap task indicate that the FEF and PPC play an important role in the disengagement of fixation.

## Conclusions

Previous work (e.g., Currie et al., 1991; Abel et al., 2002; Shafiq-Antonacci et al., 2003; Garbutt et al., 2008; Kaufman et al., 2010, 2012; Yang et al., 2013) has highlighted prosaccades as a useful biological marker for dementia. The z-score charts here, revealed that some features of prosaccades are indeed, impaired in people with AD. However, previous research had not investigated whether prosaccades change over the course of the disease. The current findings revealed that prosaccades do not necessarily deteriorate over a 12-month period in AD. People with AD vary in the specific feature of their eye-tracking impairment. We suggest that future work should not focus on a single abnormality of saccadic eye movement, but should capture a profile/pattern of abnormalities across a range of measures, both within and across the neurodegenerative diseases. We believe that eye-tracking assessments in AD will benefit from the measurement of a wide range of oculomotor parameters.

Eye tracking provides a useful methodology for monitoring changes in cognition (Shafiq-Antonacci et al., 2003; Kaufman et al., 2010, 2012; Garbutt et al., 2008). Previous work by our group and others suggest that this approach has a number of advantages over traditional methods of psychological assessment. Standard and novel experimental paradigms can be developed to evaluate theories of cognitive impairment and to dissociate between various neural networks. A careful selection of the oculomotor paradigms (which may be combined with brain imaging), have opened up opportunities for gaining new insights into the complexities of the cognitive changes in AD. Saccadic eye movements are a quantifiable and non-invasive measure of brain function that is used to identify the affected neural networks in several neuropsychiatric diseases (Broerse et al., 2001). Crawford et al. (2013) examined the saccadic eye movements of patients with Alzheimer's Disease (AD), and found abnormalities of inhibitory control that were clearly distinct from the effects in Parkinson's disease and age-matched controls. Longitudinal studies are essential to evaluate the viability of eye-tracking as a tool for monitoring the cognitive changes in the progression of dementia.

## Author contributions

TC designed the experiments; SH recruited and tested the patients and controls; SH, AD, CK analysed the eye-tracking and psychometric data; TC, AD, CK edited the manuscript. TC wrote the manuscript.

### Conflict of interest statement

The authors declare that the research was conducted in the absence of any commercial or financial relationships that could be construed as a potential conflict of interest.
